# Surgical Outcomes of Patients with Diagnostic Preoperative Monocular Occlusion in Intermittent Exotropia

**DOI:** 10.1038/s41598-020-64642-9

**Published:** 2020-05-08

**Authors:** Jin Young Lee, Ji Eun Song, Hae Ran Chang, Chul Young Choi, So Young Han

**Affiliations:** Department of Ophthalmology, Kangbuk Samsung Hospital, Sungkyunkwan University School of Medicine, Seoul, Republic of Korea

**Keywords:** Diseases, Health care, Medical research

## Abstract

We evaluated surgical outcomes of bilateral rectus (BLR) recession in patients with intermittent exotropia who underwent diagnostic monocular occlusion. Records of patients with intermittent exotropia who were preoperatively examined one-hour monocular occlusion and underwent BLR recession were reviewed retrospectively. Patients were classified into two groups based on change in exodeviation angle: responders (≥5 change in prism diopters [PD] after occlusion) or non-responders (<5 PD change after occlusion). BLR recession amount was determined by maximal angle deviation after occlusion tests in both groups. Overall follow-up period was 38.81 ± 24.09 months for non-responders (n = 106) and 38.52 ± 19.87 months for responders (n = 142) (p = 0.766). There was no difference in deviation before monocular occlusion between the two groups. Mean angle of deviation at distance (24.23 ± 6.27 PD) and near fixation (25.46 ± 6.78 PD) increased to distance (29.95 ± 6.43 PD) and near deviation (32.15 ± 6.17 PD) after occlusion in the responder group. At postoperative 1 year, surgical success rate was higher in responders (71.1%) than in non-responders (52.8%) (p = 0.003). Kaplan-Meier survival analysis revealed significantly higher surgical success in responders (p = 0.001, log-rank test). Preoperative diagnostic monocular occlusion in patients with intermittent exotropia can influence surgical outcomes by identifying the latent exodeviation angle.

## Introduction

Intermittent exotropia is the most prevalent form of manifest exodeviation. Its prevalence is about 1% in the general population^1,2^ and about 3% in Asian children^3–5^. Although many studies have shown good early surgical results, postoperative exotropic shift and recurrence of intermittent exotropia over time are common^6^.

Recurrence of exodeviation is the main consideration for intermittent exotropia. Various risk factors related to recurrence of intermittent exotropia have been reported, but there are discrepancies among studies^7–9^. One reason for recurrence is small estimation of preoperative deviation. The monocular occlusion has been suggested to identify target extent of exotropia in surgery. The occlusion test dissociates the eyes and disrupts fusional convergence that may be managing exodeviation^10,11^. Elimination of tenacious fusion leads to an increase in exotropic deviation after monocular occlusion^11–15^.

Kushner^11^ reported that patients in whom the angle deviation increased after preoperative occlusion test had better surgical outcomes and recommended surgery based on the largest angle deviation in intermittent exotropia. Hwang *et al*.^16^ argued that diagnostic monocular occlusion can help find the maximal angle of deviation not only in the divergence excess type of intermittent exotropia but also the basic type. However, only a few studies have compared the success rates and postoperative results of patients with intermittent exotropia referring to response to monocular occlusion. In this study, we aimed to evaluate surgical results of bilateral rectus (BLR) recession in intermittent exotropia patients according to diagnostic monocular occlusion results.

## Results

A total of 248 patients met the inclusion criteria; 142 (57.3%) in the responder group and 106 (42.7%) in the non-responder group. The clinical characteristics of the patients are listed in Table 1. Mean age at time of surgery and the overall follow-up time in all patients was 5.71 ± 1.94 years and 38.89 ± 21.07 months, respectively. Total amounts of bilateral rectus recession from original insertion were not significantly different between two groups. For the 142 responders, mean pre-occlusion exodeviation was 24.23 ± 6.27 PD at distance and 25.46 ± 6.78 PD at near. After one hour of monocular occlusion, mean exodeviation increased 5.73 ± 1.38 PD at distance and 6.70 ± 1.71 PD at near (p = 0.207 and p = 0.414, respectively). Stereopsis and fusion improved after surgical treatment in both groups without significant difference.

**Table 1:** Baseline characteristics of non-responders and responders.

Variables	Non-responders (n = 106)	Responders (n = 142)	p value
Age at surgery (years)	5.6 ± 1.94	5.8 ± 1.94	0.442^a^
Sex			0.709^b^
Male	60 (56.6)	77 (54.2)	
Female	46 (43.4)	65 (45.8)	
Follow-up (months)	38.81 ± 24.09	38.52 ± 19.87	0.766^a^
Cycloplegic refraction (spherical equivalent) (diopter)	−1.04 ± 1.47	−1.03 ± 1.63	0.895^a^
**Pre-occlusion deviation angle (PD)**			
Near	26.27 ± 5.16	25.46 ± 6.78	0.283^a^
Distance	25.48 ± 5.64	24.23 ± 6.27	0.105^a^
**Post-occlusion deviation angle (PD)**			
Near	27.58 ± 5.42	32.15 ± 6.17	<0.001^a^
Distance	26.29 ± 5.65	29.95 ± 6.43	<0.001^a^
**Change in deviation angle (PD)**			
Near	1.32 ± 1.86	6.70 ± 1.71	0.414^a^
Distance	0.82 ± 0.94	5.73 ± 1.38	0.207^a^
Amount of bilateral rectus recession (mm)	13.57 ± 1.79	13.93 ± 1.44	0.645^a^
Preoperative titmus stereopsis (arc seconds)	133.11 ± 126.58	116.97 ± 102.69	0.284^a^
Postoperative titmus stereopsis (arc seconds)	116.41 ± 31.79	98.45 ± 23.50	0.549^a^
Preoperative Worth-4-dot test fusion	84 (79.2)	123 (86.6)	0.122^b^
Postoperative Worth-4-dot test fusion	96 (90.6)	129 (90.8)	0.940^b^
Preoperative Bagolini Striated Glasses Test fusion	84 (79.2)	123 (86.6)	0.122^b^
Postoperative Bagolini Striated Glasses Test fusion	96 (90.6)	129 (90.8)	0.940^b^

Data are presented as mean ± standard deviation or n (%).

PD, prism diopters.

^a^Independent t-test.

^b^Pearson Chi-Square test.

Surgical outcomes were compared between the two groups (Table 2). Surgical success was achieved in 56 (52.8%) of 106 non-responders and 101 (71.1%) of 142 responders at postoperative 12 months (p = 0.003). At the last follow-up, the surgical success rate was higher in the responder group (69.2%) than in the non-responder group (50.9%) (p = 0.001). There was no overcorrection in the non-responder group and only one case (0.7%) of overcorrection in the responder group. The overcorrected patient last visited at postoperative three years and was lost to follow up thereafter. This patient’s final deviation was 8 PD esotropia, which was corrected with prism glasses. Reoperation was conducted if exotropia of 15 PD or more persisted and there was an increase in angle of deviation with decreased binocularity during postoperative follow-up. However, among them, 3 patients in non-responder group and 2 patients in responder group did not undergo reoperation because the patients and their parents were satisfied with their ocular alignment and refused to have further surgery. Significantly fewer patients in the responder group (24.4%) performed reoperation for undercorrection than in the non-responder group (43.4%) (p = 0.002).

**Table 2:** Surgical outcomes after bilateral rectus recession in non-responder and responder groups.

Variables	Non-responders (n = 106)	Responders (n = 142)	p value
Postoperative 12months			0.003^a^
Overcorrection	0 (0)	1 (0.7)	
Surgical success	56 (52.8)	101 (71.1)	
Undercorrection	50 (47.2)	40 (28.2)	
Last follow-up			0.001^a^
Overcorrection	0 (0)	1 (0.7)	
Surgical success	54 (50.9)	98 (69.2)	
Undercorrection	52 (49.1)	43 (30.3)	
Reoperation	46 (43.4)	34 (24.4)	0.002^b^

^a^Fisher’s Exact test.

^b^Pearson’s Chi-Square test.

**Figure 1:**
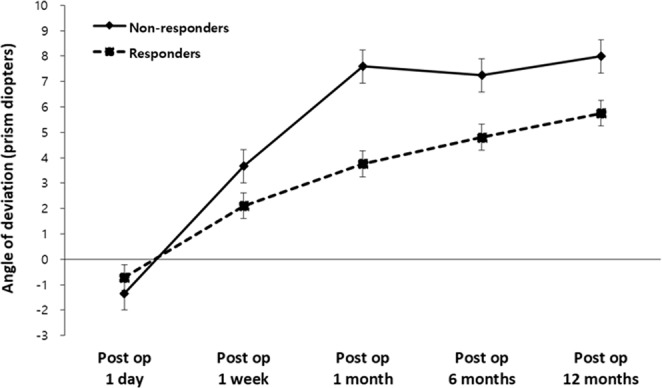
Postoperative angle of exodeviation at distance according to postoperative duration. A group x time interaction effect was found (p = 0.003) by linear mixed model analysis. With regard to angle of deviation, “−” indicates esodeviation, and “+” indicates exodeviation. *Post op* postoperative.

The effect of time on deviation from 1 day to 12 months after surgery was evaluated with linear mixed model analyses. Exotropic shift of the non-responder group was steeper than for the responder group, and the interval between the two groups increased after postoperative one week (Fig. 1). There was a significant interaction between two groups over time (p = 0.003).

**Figure 2:**
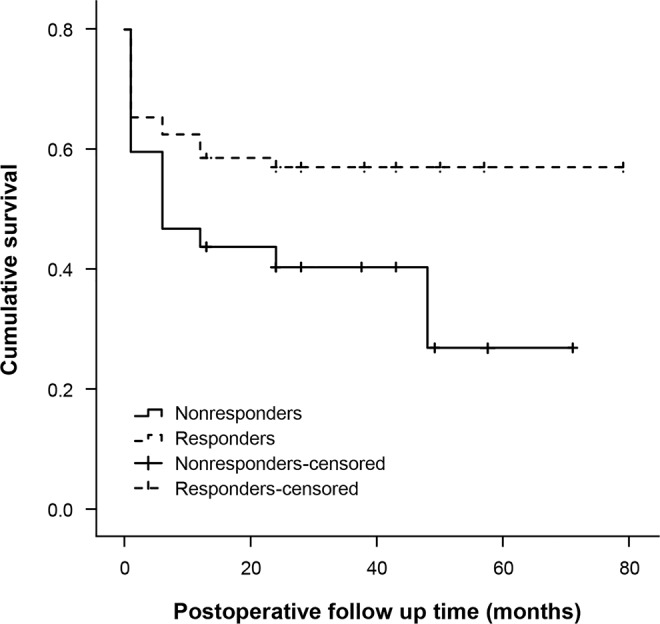
Kaplan-Meier curves for overall surgical success after bilateral rectus recession in intermittent exotropia. Differences in cumulative probability of surgical success were statistically significant (p = 0.001, log-rank test).

Kaplan-Meier survival curves for surgical success manifested that the mean survival times was 48.0 ± 24.24 months for non-responders (Fig. 2). The success rates of 12 months and 24 months for responders were 73.2 ± 3.7% and 71.3 ± 3.9%, which was significantly higher than non-responders, 54.7 ± 4.8% and 50.5 ± 6.0%, respectively. (p = 0.002, p = 0.004, respectively) Differences in cumulative surgical success rates between the two groups were statistically significant (p = 0.001, log-rank test).

## Discussion

Postoperative angle of deviation commonly increases over time in intermittent exotropia despite successful surgical outcomes in the early postoperative period. Constant exotropic shift leads to recurrence of intermittent exotropia^6–9^. Many studies have evaluated risk factors of recurrence related to surgical outcomes, and these include preoperative angle of exodeviation^17^, age at surgery^18,19^, surgical procedures^20,21^, duration of postoperative follow-up^6^, type of exotropia^15^, and stereopsis^22^. However, controversies remain.

Traditionally, the amount of surgical correction for intermittent exotropia is determined by the alternative prism cover test, where the patient fixates on an accommodative target at 1/3 meters at near and 6 meters at distance. The angle of deviation, however, may be different each examination. The success of exotropia surgery is related to the target which depends on preoperative angle, so it is important to decide the surgical volume conforming to largest angle of deviation. Postoperative initial overcorrection in intermittent exotropia is preferred considering the possibility of exotropic shift^23,24^. Although initial overcorrection naturally resolves, it remains in some cases and becomes consecutive esotropia. Patients with postoperative initial overcorrection have difficulties with unusual binocularity, for example diplopia, decreased stereopsis, and cosmetic problems^25^. Initial overcorrection can be managed with conservative method, such as close observation, alternative occlusive therapy, or prism glasses^25^. About 2.29–5.9% of patients with consecutive esotropia were reported to have considered surgical treatment for overcorrection^26–29^. Therefore, identifying the largest preoperative angle of deviation may help to prevent unnecessary undercorrection and reduce the recurrence rate. In our study, we performed BLR recession in intermittent exotropia patients based on largest angle of deviation following diagnostic occlusion and compared the outcomes with those of patients whose angle of deviation did not differ after occlusion.

A previous study reported that patients with intermittent exotropia tend to increase in the angle of misalignment when fixated on an outdoor object or under monocular occlusion^11^. Generally, the monocular occlusion test is used to differentiate pseudo-divergence excess from true divergence excess when the angle of deviation at distance is larger than that at near fixation^30^. The diagnostic occlusion test eliminates the fusional mechanism in intermittent exotropia, which increases the amount of deviation, especially at near fixation^13^. However, several studies have showed that monocular occlusion also increases the distance angle in intermittent exotropia, including basic type^12,16,31^, similar to our study.

The reason for a higher success rate in responders in the current study is unclear, but their potential fusion capacity may influence the response to surgery, and facilitate a stable surgical outcome. The patients with intermittent exotropia usually control well but intact control state is interrupted temporarily and provoke fusion loss to manifest exotropia^32,33^. Arnoldi *et al*.^34^ demonstrated that angle of deviation did not enlarge after the occlusion test at the patients in intermittent exotropia with poor fusion, but those with good control should perform preoperative occlusion test to destroy maintaining tenacious distance fusion and find inherited angle of deviation. Yildirim *et al*.^35^ reported patients with intermittent exotropia who had central fusion achieved a successful postoperative motor alignment than patients with suppression, which is similar with our study. The responder group showed a higher preoperative fusion rate (86.6%) than the non-responder group (79.2%), though it was not statistically significant. Further prospective research is needed to examine the fusional amplitude or level of control such as good, fair, and poor through cover-uncover test to investigate the potential fusional capacity in patients with intermittent exotropia.

Few studies have examined the relationship between largest deviation angle and surgical success rate. Kushner^11^ evaluated the angle of deviation in patients with intermittent exotropia while patients fixated on targets at 24 meters and 6 meters. In their study, a total of 43 of 50 patients in the study group who underwent surgery for preoperative largest angle showed successful surgical outcomes compared to 25 of 40 patients in the comparison group who underwent surgery for initial deviation at 6 meters (p < 0.001). Kim *et al*.^36^ demonstrated that patients with intermittent exotropia who received operation based on angle after 1 hour of monocular occlusion showed a higher satisfactory surgical outcome (71.4%) than patients who underwent operation based on pre-occlusion angle (62.5%), but the difference was not statistically significant. In the current study, we observed significant differences in surgical outcomes between the responder and non-responder groups, perhaps because of the different study design. We operated based on the post-occlusion angle in all patients and compared the two groups according to change in deviation after the occlusion test. Furthermore, a larger number of patients and long-term follow-up periods could have contributed to the differences in our results from those of previous studies. In non-responders, postoperative exodeviation angle increased more rapidly than for the responders, resulting in 47.2% of patient with undercorrection at one year. Surgical success was achieved in 50.9% of non-responders and 69.2% of responders at the last follow-up, which was on average at 38 months (p = 0.001). Only 0.7%(1) patients in the responder group demonstrated overcorrection. If the responder group had undergone operation according to pre-occlusion angle of deviation, they may have had demonstrated undercorrection. Therefore, the surgical success rate may have been lower^11^, and postoperative exotropic shift may have been larger and faster than expected.

Risk factors of recurrent intermittent exotropia differ among studies and remain unclear^17–22,37^. The diagnostic monocular occlusion test is noninvasive and is a simple way to weaken tenacious fusion^10,11^. Therefore, this test is beneficial to determine maximal preoperative deviation to identify potential responders and reduce the risks of recurrence. The preoperative occlusion examination should be repeated two or three times at three months intervals, as the angle of deviation may change each time and this could increase confidence in results for low-compliance patients^16^.

Our study had some limitations. It was a retrospective study and included only the basic type of intermittent exotropia operated with BLR recession. Our results can therefore not be generalized to all exotropia types. Also there were no data with fusional amplitude or levels of fusion such as good, fair, and poor. Initial postoperative target in this study was orthotropia to a small angle of esodeviation (≤5 PD), and it may have contributed to the low rate of overcorrection, although the undercorrection rate was similar to that reported in previous studies^15,20,38^. The result of some low-compliant patients may have affected to the surgical failure. Well controlled prospective studies with modified surgical tables according to response to the occlusion test and long-term follow-up are recommended.

In conclusion, preoperative monocular occlusion in patients with intermittent exotropia can considerably influence surgical outcomes after BLR recession by identifying the latent exodeviation angle.

## Methods

We conducted a retrospective study of medical records of patients diagnosed with intermittent exotropia who underwent BLR recession at Kangbuk Samsung Hospital between January 2005 and January 2016. We followed all related tenets of the Declaration of Helsinki and this work was approved by the Institutional Review Board of Kangbuk Samsung Hospital in Seoul, Korea (Approval number, KBSMC 2018–11–001). Informed consent was waived because of the minimal risk of this work. Exclusion criteria were patients with less than 1 year of postoperative follow-up, A or V patterns, presence of with lateral incomitances (changes ≥10 prism diopters [PD] in lateral gaze compared with the primary position) or oblique muscle overactions, previous strabismus surgery, strabismus secondary to congenital malformations or neurologic diseases, paretic or restrictive strabismus, sensory exotropia, or infants and children under three years of age who were too young to cooperate with examination.

All intermittent exotropia patients underwent full ophthalmologic examination before the operation. The examination involved best-corrected visual acuity, full cycloplegic refraction, and fundus examination. Angles of deviation were measured by simultaneous and alternate prism cover tests, and ductions and versions were evaluated. Stereopsis was assessed by the Titmus test and good stereopsis was defined as less than 100 seconds of arcs. Bagolini striated glasses and Worth-4-dot tests were performed to evaluate binocularity. Angle of deviation, Bagolini striated glasses and Worth-4-dot tests were assessed at 6 meters (distance) and 33 centimeters (near). Angle of deviation was measured before and 1 hour after monocular occlusion and was repeated on at least three visits before surgery to rule out progressing exotropia if the degree of exotropia has increased. When one’s angle of deviation increased continuously, we regarded as progressing intermittent exotropia and postponed the operation until there were no more changes, confirming with monocular occlusion test at least three times in the same manner. Before removing the occlusion, the patient was asked to close both eyes. The ophthalmologist masked the non-occluded eye with an occluder to ensure that the patient did not peek around and regain fusion. Then an assistant gently removed the occlusion patch. After the ophthalmologist reconfirmed that the patient’s eye was still closed, the patient was requested to open the eyes and the occluder was moved to the other eye to measure the angle of deviation. Patients with an increase in exodeviation of greater than 5 PD after occlusion were defined as responders and those angle of deviations which were unchanged or showed less than 5 PD in change after occlusion were defined as non-responders. Under general anesthesia, all patients were operated on by one strabismus specialist (HR.C.) for the largest angle of deviation assessed using Park’s table^39^. Postoperative angle deviation was assessed one day, one week, one month, six months, and 12 months after surgery, with final alignment performed at the last visit. All extraocular movement assessments were conducted by the same strabismus specialist (HR.C.) and were repeated to increase reliability. Surgical success was defined as esotropic deviation less than 5 PD or exotropic deviation less than 10 PD with good stereopsis.

Statistical analyses were done using IBM SPSS Statistics (V.24.0.0, IBM Corp, Armonk, NY, USA) software. Continuous data are represented as mean ± standard deviation (SD). Preoperative and postoperative variables were compared using independent sample t test. Categorical variables are showed as ratios and were compared with the Pearson χ^2^ test or Fisher’s exact test. Linear mixed models were applied to analyze of repeated postoperative angle deviation at different time periods. Kaplan-Meier survival analysis and log-rank tests were conducted to compare the cumulative probabilities of success over time between the two groups. Surgical failure at any postoperative follow-up time was censored. Probability values less than 0.05 were considered significant.

## Data Availability

All data generated or analysed during this study are included in this published article.
